# Associations between study questionnaire-assessed need and school doctor-evaluated benefit of routine health checks: an observational study

**DOI:** 10.1186/s12887-021-02810-0

**Published:** 2021-08-16

**Authors:** Kirsi Nikander, Elina Hermanson, Tero Vahlberg, Minna Kaila, Tuire Sannisto, Silja Kosola

**Affiliations:** 1 City of Helsinki, Department of Social Services and Health Care, School and Student Health Care, P.O. Box 6100, FI-00099 Helsinki, Finland; 2 Doctoral School in Health Sciences, Doctoral Program in Population Health, University of Helsinki, Helsinki, Finland; 3 Department of General Practice and Primary Health Care, University of Helsinki and Helsinki University Hospital, Helsinki, Finland; 4 Pikkujätti Medical Center for Children and Youth, Annankatu 32, 00100 Helsinki, Finland; 5 Faculty of Medicine, Department of Clinical Medicine, Biostatistics, University of Turku, Kiinamyllynkatu 10, FI-20520 Turku, Finland; 6 University of Helsinki, Public Health Medicine, P.O. Box 63, FI-00014 Helsinki, Finland; 7 City of Tampere, Social and Health Care Services, Services for Children, Youth and Families, Naulakatu 2, FI-33101 Tampere, Finland; 8 Pediatric Research Center, New Children’s Hospital, Helsinki University Hospital, University of Helsinki, HUS, P.O. Box 705, Biomedicum 2 C, FI-00029 Helsinki, Finland

**Keywords:** Children, Questionnaires, Health check, School doctor, School health services

## Abstract

**Background:**

In Finland, school doctors examine all children at predetermined ages in addition to annual health checks by school nurses. This study explored the association of study questionnaire-assessed need for and school doctor-evaluated benefit of routine health checks conducted by doctors.

**Methods:**

Between August 2017 and August 2018, we recruited a random sample of 1341 children in grades 1 and 5 (aged seven and eleven years, respectively) from 21 elementary schools in four Finnish municipalities. Children mainly studying in special education groups or whose parents needed an interpreter were excluded. School nurses performed their health check as usual. Parents, nurses, and teachers then completed study questionnaires that assessed the concerns of parents, school nurses, and teachers regarding each child’s physical, mental and social health. Doctors, blinded to the responses, routinely examined all the children. The primary outcome measures were (1) the need for a health check based on the study questionnaires and (2) the benefit/harm of the appointment as estimated by the doctors according to predetermined criteria, and (3) the patient-reported experience measures (PREMs) of benefit/harm of the appointment as estimated by the parents and children. We compared the need for a health check with the doctor-evaluated benefit using multilevel logistic regression.

**Results:**

The participation rate was 75.5 %. According to all questionnaires, 20–25 % of the 1013 children had no need for a health check. The doctors regarded 410 (40.6 %) and the parents 812 (83.4 %) of the appointments as being beneficial. Respondents rarely reported harm. The children who were classified as needing a health check more often benefitted from the health check (assessed by the doctor) than children with no need for one (OR 3.53; 95 % CI 2.41–5.17).

**Conclusions:**

The need for a health check is an important predictor of school-doctor evaluated benefit of the health check. This approach could allow school doctors to allocate time for the children who need them most.

**Trial registration:**

ClinicalTrials.gov, Identifier NCT03178331, registration June 6th 2017.

**Supplementary Information:**

The online version contains supplementary material available at 10.1186/s12887-021-02810-0.

## Background

School health services exist in at least 102 countries [1] and provide a unique opportunity to identify and help children at risk of long-lasting physical, mental and social problems. Even in high-income nations, poorer children are at increased risk of adverse health outcomes [2]. One solution to this problem could be school-based health centers that can advance health equity [3]. Large-scale interventions by school health services may be more cost-effective than individual screening procedures [4, 5]. The interventions should focus on the individual, family, and community [6].

Substantial gaps exist in the evidence supporting many preventive care recommendations such as behavioral counceling and screening which are often included in health checks [9]. According to a review of research published in English from 1943 to 1995, yearly physical examinations had no value in detecting important pathologic conditions in adolescents [10]. The diagnostic accuracy of blood pressure measurements among asymptomatic children and the benefits and harm of screening for adolescent idiopathic scoliosis are unclear [11–13]. In the Netherlands, no significant differences were found in the detection of children’s overweight, visual disorders or psychosocial problems between doctors’ assistants, nurses, and doctors [14]. A German study suggested that health checks by physicians at school entry could be reduced by targeting them at children who are at risk of a severe developmental disorder [15]. However, a recent review found evidence supporting the implementation of anxiety prevention programs, asthma education, and vision screening [16].

In Finland, the public preventive health care of children and young people is legally bound to offer routine general health checks by nurses and doctors in addition to appointments related to special health care needs [7]. Prior to the age of 6 years, well child clinics offer children fifteen general health checks by nurses and five health checks that also involve a doctor. Despite the lack of robust evidence supporting health checks of asymptomatic school children, school nurses conduct annual health checks of children aged 7–15 years. In grades one, five, and eight (at ages seven, eleven, and fourteen years, respectively) parents are invited to participate in the extensive health checks. During these extensive health checks, both the nurse and the doctor check the children regardless of previously identified children’s health risks. The roles of school nurses and school doctors are described in Table 1 of the study protocol [8]. The major differences between nurses and doctors are that doctors evaluate the status of the child more extensively than nurses (auscultation of heart and lungs, examination of testicles, psychiatric and neurologic status, diagnostics and differential diagnostics), and write prescriptions and referrals to secondary care when needed. Local authorities regulate the execution of school doctors’ health checks at predetermined ages, which restricts the use of doctors’ expertise in schools. School doctors have insufficient time for children in other age groups, for multidisciplinary work, and for the treatment of school-related health problems.

**Table 1:** Baseline characteristics of participating 1013 children and 14 doctors

	Grade 1		Grade 5		Total		
**Children**	506 (50.0)		507 (50.0)		1013 (100.0)		
Age	7.66 (7.39–7.91)		11.38 (11.13–11.66)		10.57 (7.66–11.39)		
Sex							
Female	248 (49.0)		247 (48.7)		495 (48.9)		
Male	258 (51.0)		260 (51.3)		518 (51.1)		
**Doctors**							
Sex							
Female					12 (86)		
Male					2 (14)		
Specialist degree					7 (50)		
MD in a health center including school doctor’s work					6 (43)		
Full-time school- and/or well child clinic doctor					8 (57)		
Work experience as a MD in a health center (years)					6.75 (1–12)		

Data are expressed as median (interquartile range) or n (%). Data were 100 % complete.

*MD* medical doctor

It is not known if the health check by the school nurse is sufficient for elementary school children. The primary aim of this study was to explore the association of study questionnaire-assessed need for and school doctor-evaluated benefit of routine general health checks of elementary school children in grades 1 and 5 (at ages seven and eleven years, respectively).

## Methods

### Study design

**Fig. 1:**
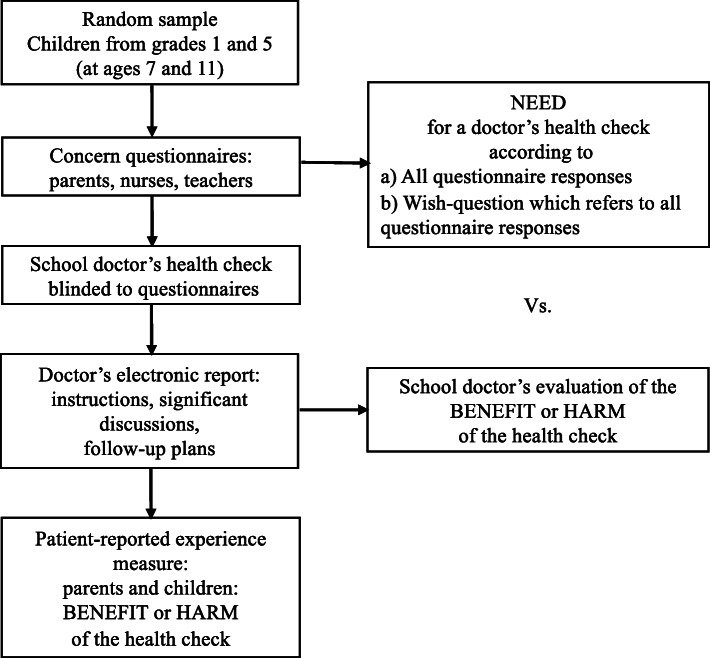
Study design

The study protocol for this observational study is available online [8]. The school nurses performed their health check as usual. Before the health check conducted by a school doctor, parents, school nurses, and teachers reported their concerns regarding each child. These concerns constituted the need for a health check. After the health check, this need was compared with the benefit or harm of the health check as assessed by the doctors according to predetermined criteria (Fig. 1). In addition, the parents and children filled in patient-reported experience measures (PREMs) of the benefit or harm. The study was undertaken in 2017–2018 in four municipalities in Finland. In one of the municipalities (Helsinki) doctors worked exclusively in schools, whereas in the three other municipalities (Tampere, Kerava and Kirkkonummi), doctors regularly visited schools but some also provided services in well child-clinics and health centers.

### Participants

In Helsinki, six school doctors consented to participate in the study and they chose schools from different socioeconomic areas in the city. In Tampere, Kirkkonummi and Kerava, the medical directors recruited doctors with varying education and work experience and schools from different socioeconomic areas. All the schools were Finnish language elementary schools. The school nurses and teachers were recruited from the participating schools.

Between August 22, 2017 and August 31, 2018, we recruited a random sample of 1341 eligible children from the participating schools. Exclusion criteria were children mainly studying in special education groups and the parents’ need for an interpreter. The study nurse assigned all eligible children a computer-generated random number. The first 30 children in each school and their guardians were asked to participate. If more than five families declined, more children were recruited from the random order list.

### Procedures

Parents are invited to participate in the extensive health checks in grades 1 and 5. Before the health check by nurses, the nurses receive background information of the child and family from the questionnaires provided by the National Institute for Health and Welfare (parent 1st and 5th grade), child 5th grade and teacher (some schools). These questionnaires have been developed to guide discussion. School nurses performed their health check as usual. Parents, school nurses, and teachers then completed study questionnaires which assessed the concerns of the parents, school nurses, and teachers regarding each child’s physical and mental health and the well-being of the whole family. One question from the Strengths and Difficulties Questionnaire (SDQ), “Overall, do you think that your child has difficulties in one or more of the following areas: emotions, concentration, behaviour or being able to get on with other people?” was modified into four separate questions [17–20]. The questionnaires also included questions regarding the child’s growth, physical well-being, eating, sleeping, learning, school absenteeism, and the well-being of the whole family, based on previous evidence [21–26], and the clinical knowledge of the research group. One final question inquired whether the respondent wished for a school doctor’s assessment of these or some other concerns.

All school doctors performed their health checks as usual blinded to the questionnaire responses. The doctors had access to routine background information and patient records. After each health check, the doctors filled in an electronic report that included any interventions undertaken during the health check and evaluated the benefit or harm of the health check. The parents, and children filled in patient-reported experience measures (PREMs) of the benefit or harm.

### Outcomes

The primary outcome measures were (1) the need for a health check by a doctor based on the questionnaires and (2) the benefit/harm of the health check as estimated by the doctors, according to predetermined criteria, and (3) the PREMs of the benefit/harm of the health check as estimated by the parents and children.

We assessed the need for a health check by a doctor based on the questionnaires completed by the parents, teachers, and nurses. Responses were categorized into three groups: (1) “No need for a health check by a doctor”, (2) “Needs a health check by a doctor”, and (3) “Consulting with a nurse or doctor may be sufficient”. The categorization of questionnaire responses is described in the study protocol [8]. Missing responses to individual questions were considered to indicate no concern or no wish for a school doctor’s assessment of the concerns. An empty questionnaire was reported as missing and excluded from the analyses.

Participating doctors assessed the benefit or harm of each health check according to criteria described in Table 2 of the study protocol [8]. The doctors reported quite a lot or a great deal of benefit if they staged any significant interventions based on predetermined criteria. The doctors reported “Only a little benefit” if the nurse could have replaced the doctor. The doctors reported harm if the interaction was unsatisfactory, or if they suspected no progress in care or refusal of school doctor services in the future. The parents and children rated how beneficial or harmful they found the school doctor’s examination without predetermined criteria. The English versions of the PREMs are provided in Additional files 5 and 6 of the study protocol [8]. In contrast to the study protocol and to increase clarity, we reported the doctors’ and parents’ evaluations of benefit and harm separately.

**Table 2:** Reason for non-participation of children

	Non-participation		
	Grade 1 (*n* = 139)n (%)	Grade 5 (*n* = 189)n (%)	Total (*n* = 328)n (%)
Child or parent refused to participate	96 (69.1)	149 (78.8)	245 (74.7)
No show	29 (20.9)	15 (7.9)	44 (13.4)
Child alone with no consent forms	7 (5.0)	22 (11.6)	29 (8.8)
Moved or changed school	3 (2.2)	1 (0.5)	4 (1.2)
Other reason	2 (1.4)	1 (0.5)	3 (0.9)
No reason indicated	2 (1.4)	1 (0.5)	3 (0.9)

The questionnaires with a free description of concern require time-consuming individual analysis. Therefore, we also conducted an exploratory analysis of the need for a health check by a doctor according to the final ‘wish question’ of the study questionnaires which refers to all the other concern questions of the study questionnaires. For parents, the question was: “Do you wish to speak with the school doctor about these concerns or some other concern related to the child’s well-being?” For nurses and teachers, the question read: “Do you wish the school doctor to address these concerns or some other concern related to the well-being of the pupil?” The response options were: (1) “Yes”, (2) “No”, or (3) “I don’t know”. Responses to the wish question were categorized into three groups as follows: (1) “Yes” = “Needs a health check by a doctor”, (2) “No” = “No need for a health check by a doctor”, and (3) “I don’t know” = “Consulting with a nurse or doctor may be sufficient”. Missing study questionnaire responses were treated as missing values and excluded from the analyses.

### Statistical analyses

According to power calculations, 450 children were needed from both grades to detect a 20 % difference (25 % vs. 45 %) in the benefit between children in need of and children with no need for a health check by a doctor [8]. Frequencies with percentages and medians with interquartile ranges were used as descriptive statistics.

The intrarater and interrater reliability of the evaluation of need were assessed with the kappa coefficient. KN evaluated the need for a health check by a doctor from the whole data and 200 randomly selected cases for intrarater reliability. To assess interrater reliability, SK evaluated the data from 200 randomly selected children. SK repeated the evaluation of the same 200 children to assess her intrarater reliability. TS resolved any discrepancies. Only questionnaires with a free description of concerns required manual analysis. Otherwise, the need was assessed using a formula according to predetermined criteria [8].

The association of the need for a health check by a doctor (based on the concerns of parents, teachers and school nurses) and the benefit of the health check by a doctor (assessed by the doctor) was analyzed using multilevel logistic regression analysis to account for the clustered nature of the data. Multilevel logistic regression models were used with child at level one, school at level two, doctor at level three, and municipality at level four. Results were expressed using odds ratios (OR) with 95 % confidence intervals (CI). Analyses were also conducted separately for children in grades one and five because the outcome may differ in different age groups. SAS 9.4 System for Windows (SAS Institute Inc., Cary, NC) was used for multilevel modeling. Other analyses were performed using IBM SPSS Statistics 25.0 for Windows (IBM Corp., Armonk, NY). P-values less than 0.05 were considered statistically significant.

## Results

From 1341 eligible children, 1013 (75.5 %) participated in the study. In total, 506 first graders (78.4 %) and 507 fifth graders (72.8 %) and their parents, 14 doctors, 31 nurses and 105 teachers from 21 schools participated in the study. Half of the doctors held some specialist degree and over half worked full-time in schools and/or well-child clinics (Table 1). The numbers of missing or late questionnaires, electronic reports by doctors, and PREMs of parents and children are presented in Additional file 1. The reasons for non-participation of children are collated in Table 2.

According to all questionnaire responses, 212 children (20.9 %) had no need for a health check by a school doctor (Table 3). Parents, nurses, and teachers respectively provided 542 (54.4 %), 563 (58.2 %), and 305 (37.4 %) free descriptions of their concerns, respectively. The kappa measures of agreement for inter- and intrarater reliability of the questionnaires were over 0.7 (good) and 0.8 (excellent), respectively.

**Table 3:** Need for school doctor’s health check according to all study questionnaire responses

Respondents		Grade 1,n (%)	Grade 5,n (%)	Total,n (%)
Parents				
	Need-	183 (36.8)	212 (42.4)	395 (39.6)
	Need+	314 (63.2)	288 (57.6)	602 (60.4)
Nurses				
	Need-	228 (47.6)	224 (45.7)	452 (46.6)
	Need+	251 (52.4)	266 (54.3)	517 (53.4)
Teachers				
	Need-	252 (63.6)	259 (61.8)	511 (62.7)
	Need+	144 (36.4)	160 (38.2)	304 (37.3)
Parents and nurses^a^				
	Need-	126 (24.9)	131 (25.8)	257 (25.4)
	Need+	380 (75.1)	376 (74.2)	756 (74.6)
Parents, nurses, and teachers^a^				
	Need-	106 (20.9)	106 (20.9)	212 (20.9)
	Need+	400 (79.1)	401 (79.1)	801 (79.1)

*Need-* “No need for a doctor’s health check”. *Need+* “Needs a doctor’s health check” and “Consulting a nurse/doctor may be sufficient” combined. ^a^Need- indicates that none of the respondents had Need+. Need + indicates that at least one of the respondents had Need+.

The doctors evaluated 410 (40.6 %) of the health checks as being beneficial. The parents and children respectively reported benefit from 812 (83.4 %) and 598 (60.3 %) health checks (Table 4). In 42 cases, the doctors considered the health check beneficial but the parents disagreed. In 113 cases, the doctors evaluated the appointments as being beneficial purely due to the instructions provided or the discussion held. In 14 of these cases, the parent disagreed with the doctors’ evaluation. All evaluations of benefit and harm are presented in Additional file 2.

**Table 4:** Evaluation of the benefit of a doctor’s health check by the doctors, parents, and children

Respondents		Grade 1, n (%)	Grade 5, n (%)	Total, n (%)
Doctors				
	Benefit-	313 (62.1)	287 (56.7)	600 (59.4)
	Benefit+	191 (37.9)	219 (43.3)	410 (40.6)
Parents				
	Benefit-	75 (15.2)	87 (18.2)	162 (16.6)
	Benefit+	420 (84.8)	392 (81.8)	812 (83.4)
Children				
	Benefit-	173 (35.0)	220 (44.3)	393 (39.7)
	Benefit+	321 (65.0)	277 (55.7)	598 (60.3)

Benefit+ = “Quite a lot of benefit” and “A great deal of benefit” combined. Benefit- = “Only a little benefit”, “No benefit or harm”, “Only a little harm”, “Quite a lot of harm” and “I don’t know” combined. The option “Only a little benefit” was also included in Benefit- because this group comprised findings and instructions that the school nurse could have provided.

Doctors reported harm from six appointments. In all of these cases, they felt that the interaction had failed. Parents reported harm from three appointments, two of which were due to failed interaction or an unnecessary check of a healthy child. One parent’s report of harm was an error, because it was based on the argument of receiving advice and help for the problem. Twelve children reported harm because of pain (vaccination or planned blood test), their dislike of being touched or having to undress, or because of being bored.

The children who were classified as needing a health check more often benefitted from it (assessed by the doctor) than those with no need for a health check (OR 3.53; 95 % CI 2.41–5.17) (Table 5). In total, doctors evaluated 42 health checks as beneficial despite no need for a health check based on questionnaires by parents, nurses, and teachers. Two of these 42 children had a minor finding (gliding testis) that is usually only recognized by physicians. In total, doctors evaluated 431 health checks as non-beneficial despite need for a health check based on questionnaires by parents, nurses, and teachers. In 264 of these 431 cases the doctor reported “Only a little benefit”, in 161 cases “No benefit or harm”, and in 6 cases harm.

**Table 5:** Association of need for and doctor-evaluated benefit of the doctor’s health check; multilevel logistic regression analysis

	**Grade 1**				**Grade 5**				**Total**			
		Benefit+				Benefit+				Benefit+		
Need	N	n (%)	OR (95 % CI)	*P*-value	N	n (%)	OR (95 % CI)	*P*-value	N	n (%)	OR (95 % CI)	*P*-value
**Parents**												
Need-	182	37 (20.3)	1		212	69 (32.6)	1		394	106 (26.9)	1	
Need+	313	152 (48.6)	3.90 (2.50–6.08)	< 0.0001	287	148 (51.6)	2.34 (1.59–3.45)	< 0.0001	600	300 (50.0)	2.94 (2.20–3.92)	< 0.0001
**Nurses**												
Need-	227	67 (29.5)	1		224	70 (31.3)	1		451	137 (30.4)	1	
Need+	250	112 (44.8)	2.03 (1.36–3.03)	0.0006	265	140 (52.8)	2.55 (1.72–3.78)	< 0.0001	515	252 (48.9)	2.20 (1.66–2.92)	< 0.0001
**Teachers**												
Need-	252	90 (35.7)	1		259	93 (35.9)	1		511	183 (35.8)	1	
Need+	144	66 (45.8)	1.62 (1.03–2.53)	0.0365	159	86 (54.1)	2.17 (1.42–3.31)	0.0004	303	152 (50.2)	1.82 (1.34–2.47)	0.0001
**Parents and nurses**^a^												
Need-	125	25 (20.0)	1		131	32 (24.4)	1		256	57 (22.3)	1	
Need+	379	166 (43.8)	3.54 (2.12–5.90)	< 0.0001	375	187 (49.9)	3.19 (2.01–5.07)	< 0.0001	754	353 (46.8)	3.26 (2.31–4.60)	< 0.0001
**Parents, nurses, and teachers**^a^												
Need-	105	18 (17.1)	1		106	24 (22.6)	1		211	42 (19.9)	1	
Need+	399	173 (43.4)	4.11 (2.32–7.28)	< 0.0001	400	195 (48.8)	3.30 (1.98–5.51)	< 0.0001	799	368 (46.1)	3.53 (2.41–5.17)	< 0.0001

Need- = No need for a doctor’s health check. Need + includes categories “Needs a doctor’s health check” and “Consultation of nurse/doctor may be sufficient” combined. ^a^Need- indicates that none of the respondents had Need+. Need + indicates that at least one of the respondents had Need+. Benefit + is “Quite a lot or more benefit”. Benefit- includes responses “Only a little benefit”, “No benefit or harm”, “Only a little harm”, “Quite a lot of harm” and “I don’t know” combined. *OR* Odds ratio, *CI* Confidence interval.

In the exploratory analysis, the single wish question proved as valuable as using all the questionnaire responses for determining the need for a health check by the school doctor. According to the responses to the wish question, 257 children (25.4 %) had no need for a doctor’s appointment (Additional file 3). In this analysis, the children in need of a health check also more often benefitted (assessed by the doctor) from the health check than those with no need for one (OR 3.60; 95 % CI 2.53–5.11) (Additional file 4).

## Discussion

The children who were classified as needing a health check more often benefitted from the health check than children with no need for a health check. In this study, 20–25 % of the participating children had no need for a health check by a school doctor. Doctors evaluated two-fifths and parents four-fifths of health checks as being beneficial. All respondents rarely reported harm.

Nurses wished that the school doctor would address their concerns regarding over half of the children in grades one and five, which is surprisingly high. Children in other grades rarely meet a school doctor even when they have special health care needs, for example, related to overweight or obesity [27]. However, many health problems would be most efficiently detected and managed in the school [28, 3]. Furthermore, nurses may be unaware of the concerns of parents and teachers regarding children in other grades since they often meet children alone during the annual health checks between the extensive health checks in grades 1 and 5. The value of these health checks by school nurses without collaboration with parents and teachers is questionable.

Parents considered most health checks as beneficial. This may reflect their appreciation of the doctor thoroughly checking their child, as well as the opportunity to have a discussion with a doctor. On the other hand, the doctors reported benefit from 42 health checks that the parents considered of minor value. In these cases, the doctor may have regarded some intervention important although it had no current value to the parent.

One-fifth of the children with no need for a health check by a school doctor benefitted from it according to the doctor. Several explanations for under-reporting of some concerns prior to the health check are plausible: (1) the parents’ concerns may have changed, even within a short time after filling in the questionnaire; (2) the parents may remember or express their concerns only when meeting a professional; (3) the parents and teachers may be unaware of the doctor’s role in school health services and thus expect no help from the doctor for psychosocial or learning problems; and (4) the doctors may offer prescriptions, for example, for skin conditions and allergies that are unessential at the time but likely to be necessary in the future.

Children rarely had findings that require a doctor’s expertise to be recognized. In all, 42 children who had no questionnaire-based need for a health check, still benefitted of the health check. Two of these children had a minor finding (gliding testis) that is usually only recognized by physicians. Hutson and coworkers recommended the screening of all boys for acquired undescended testis at school entry [29]. In Finland, children are already thoroughly examined for this condition as well as other similar conditions such as heart murmurs [30, 31] in health checks at the age of four years, in addition to earlier health checks in well-child clinics. These results are in line with a review in which annual physical examinations had no value in detecting important pathologic conditions in adolescents [10].

Over half of the children with need for a health check gained no benefit from it according to the doctor. Most of these cases belonged to the categorization “Only a little benefit” which comprised findings and instructions that the school nurse could have provided. Several other factors may explain this finding. The concerns may have changed, even within a short time after filling in the questionnaire. The doctors may have been unaware of the teachers’ concerns and unable to target interventions. Parents and teachers may disagree about their concerns regarding a child. Despite significant concerns the family and/or child may have been referred to appropriate specialists already earlier and required no interventions from the school doctor.

Our study has several strengths. The study was conducted in a “real world” setting of children’s regular health checks with a high participation rate. We reduced information bias by similar training of the participating doctors, nurses, and teachers and by blinding the doctors to the study questionnaire responses. The researchers assessed the need for a health check by a doctor without knowing the evaluations of the benefit provided by the doctors and parents. The study included schools and professionals from different municipalities and socioeconomic areas, which increases the generalizability of the results. The questionnaires were easy to understand and could be completed within a few minutes. Using only the ‘wish question’ to determine the need for a health check by a doctor was especially feasible, since the considerable amount of free descriptions of concern became rudundant. However, the ‘wish question’ refers to all the other concern questions of the study questionnaires and thus cannot be used alone. In practice, the questionnaires could be provided digitally throughout the school year, which could improve the timeliness of the health check. School doctors could also utilize all the questionnaire responses (multi-informant approach) to target interventions during the health check, unlike in the present research setting.

The study also had some limitations. A randomized controlled trial was inconceivable because school health care in Finland is legally defined. Recruiting a control group that would undergo no health checks was impossible. However, the doctors performed their health checks as usual for all children and were blinded to the study questionnaire responses. Although the participation rate in this study was high, selection bias may have occurred, since a quarter of invited families refused to participate. Non-participants may have been families who had the most stressors in their life. The frequency of missing questionnaires from parents and nurses was low, but about one-fifth of the teachers’ questionnaires were missing. However, the use of a multi-informant approach reduced the impact of missing questionnaires. The participating doctors were not randomly selected. However, doctors with varying education and work experience from different cities participated in the study and only three doctors’ evaluations of benefit or harm were missing. Children mainly studying in special education groups and children whose parents needed an interpreter were excluded. In these vulnerable groups, involving the school doctor is plausibly beneficial to provide information and ascertain adequate health and social care contacts and rehabilitation services. Information bias may have occurred when doctors evaluated the benefit of the health check. Although the doctors followed previously defined criteria when assessing benefit, subjectivity was impossible to eliminate. We accounted for this in the statistical analyses by using multilevel logistic regression and included different doctors as one of the four covariates.

We focused on a surrogate outcome of benefit that the doctors evaluated immediately after each health check. Surrogate end points can fail to predict a true clinical outcome [32]. General health checks in adults have been found to increase the number of new diagnoses but failed to reduce morbidity and mortality [33]. However, if the school doctor conducted no interventions during the health check, substantial benefit would be improbable. Even the health checks considered beneficial should be appraised critically as several reasons may diminish the actualized benefit. Families may neglect the suggested tests and treatment plans. The intensity of the interventions may be insufficient to affect both the school and the family environment [34]. Despite the preventive health care system in Finland, Häkkänen and coworkers demonstrated that obesity increased and obese children remained obese during the primary school years [35]. Furthermore, other pathways may affect any clinical outcome. Parents may contact doctors or other professionals outside the school health services. Unpredictable adverse life events may also occur after the health check.

Although organizational models of school health services differ globally [1, 28, 36, 37], this proposed approach of concern-based health checks by school doctors may be useful internationally. In the future, this method of assessing children’s need for a health check by a doctor could be tested digitally in a country where general health checks by doctors are non-obligatory. A multidisciplinary approach in co-operation with the family to find the most suitable way to reduce concerns detected by this method may be valuable. The ability to coordinate effective preventive care requires multidisciplinary work related to poverty, educational outcomes, the healthiness of social and physical environments, and healthy lifestyle choices [6, 38]. Doctors should have time to participate in multidisciplinary work within the school, and cooperate with family counselling, child-protection services, and secondary care more often than is currently feasible. Children with special health care needs require more services than their peers [39].

## Conclusions

The children who were classified as needing a health check more often benefitted from the health check than children with no need for a health check. The need for a health check is an important predictor of school-doctor evaluated benefit of the health check. If unnecessary routine general health checks were omitted, school doctors could allocate more time for children and families in greatest need, regardless of the grade. These findings can be used in future studies and policymaking to enhance equity in school health services.

## Supplementary Information

**Additional file 1: **Eligible study questionnaires of parents, nurses, and teachers, doctors' electronic reports and parents' and children's PREMS

**Additional file 2: **Evaluation of the benefit or harm of a doctor’s health check by the doctors, parents, and children

**Additional file 3:** Need for a doctor’s health check according to the wish question of the study questionnaires

**Additional file 4: **Association of the response to the wish question and benefit of the doctor’s health check; multilevel logistic regression

**Additional file 5: **STROBE checklist

## Data Availability

The datasets generated and analyzed during the current study are not publicly available due to restrictions that applied under the license for the study but are available from the corresponding author KN and MK on reasonable request.

## Abbreviations

SDQ: Strengths and Difficulties Questionnaire
PREM: Patient-reported experience measure
OR: Odds ratio
CI: Confidence interval

## References

[1] Baltag V, Pachyna A, Hall J (2015). Global Overview of School Health Services: Data from 102 Countries. Health Behav Policy Rev.

[2] Spencer N, Raman S, O’Hare B, Tamburlini G. Addressing inequities in child health and development: towards social justice. BMJ Paediatr Open. 2019;3. 10.1136/bmjpo-2019-000503. 10.1136/bmjpo-2019-000503 PMC6688679 31423469

[3] Knopf JA, Finnie RKC, Peng Y, Hahn RA, Truman BI, Vernon-Smiley M (2016). School-Based Health Centers to Advance Health Equity: A Community Guide Systematic Review. Am J Prev Med.

[4] Sheehan P, Sweeny K, Rasmussen B, Wils A, Friedman HS, Mahon J (2017). Building the foundations for sustainable development: a case for global investment in the capabilities of adolescents. The Lancet.

[5] Michaud P-A, Namazova-Baranova L, Weber M, Ambresin A-E (2018). Effective School Health Service: A Response to Adolescent Health Needs in Europe. J Pediatr.

[6] Forrest CB, Riley AW (2004). Childhood origins of adult health: a basis for life-course health policy. Health Aff Proj Hope.

[7] Ministry of Social Affairs and Health. Valtioneuvoston asetus neuvolatoiminnasta, koulu- ja opiskeluterveydenhuollosta sekä lasten ja nuorten ehkäisevästä suun terveydenhuollosta (The government decree on maternity and child health clinic services, school and student health services and preventive oral health services for children and youth). 2011. http://www.finlex.fi/fi/laki/smur/2011/20110338. Accessed 28 Dec 2019.

[8] Nikander K, Kosola S, Kaila M, Hermanson E (2018). Who benefit from school doctors’ health checks: a prospective study of a screening method. BMC Health Serv Res.

[9] Moyer VA, Butler M (2004). Gaps in the Evidence for Well-Child Care: A Challenge to Our Profession. Pediatrics.

[10] Stickler GB (2000). Are Yearly Physical Examinations in Adolescents Necessary?. J Am Board Fam Pr.

[11] Grossman DC, Curry SJ, Owens DK, Barry MJ, Davidson KW, Doubeni CA (2018). Screening for Adolescent Idiopathic Scoliosis: US Preventive Services Task Force Recommendation Statement. JAMA.

[12] Płaszewski M, Grantham W, Jespersen E. Mapping the evidence of experiences related to adolescent idiopathic scoliosis: a scoping review protocol. BMJ Open. 2019;9. 10.1136/bmjopen-2019-032865. 10.1136/bmjopen-2019-032865 PMC6886945 31753899

[13] Gartlehner G, Vander Schaaf EB, Orr C, Kennedy SM, Clark R, Viswanathan M. Screening for Hypertension in Children and Adolescents: Updated Evidence Report and Systematic Review for the US Preventive Services Task Force. JAMA. 2020;324(18):1884–95. 10.1001/jama.2020.11119 33170247

[14] Bezem J, Theunissen M, Kamphuis M, Numans ME, Buitendijk SE, Kocken P (2016). A Novel Triage Approach to Identifying Health Concerns. Pediatrics.

[15] Führer A, Wienke A, Wiermann S, Gröger C, Tiller D (2019). Risk-based approach to school entry examinations in Germany – a validation study. BMC Pediatr.

[16] Levinson J, Kohl K, Baltag V, Ross DA. Investigating the effectiveness of school health services delivered by a health provider: A systematic review of systematic reviews. PLOS ONE. 2019;14. 10.1371/journal.pone.0212603. 10.1371/journal.pone.0212603 PMC6561551 31188826

[17] Goodman R (1999). The extended version of the Strengths and Difficulties Questionnaire as a guide to child psychiatric caseness and consequent burden. J Child Psychol Psychiatry.

[18] Goodman R (2001). Psychometric Properties of the Strengths and Difficulties Questionnaire. J Am Acad Child Adolesc Psychiatry.

[19] Goodman A, Goodman R (2011). Population mean scores predict child mental disorder rates: validating SDQ prevalence estimators in Britain: SDQ prevalence estimators. J Child Psychol Psychiatry.

[20] Borg A-M, Salmelin R, Joukamaa M, Tamminen T (2014). Cutting a Long Story Short? The Clinical Relevance of Asking Parents, Nurses, and Young Children Themselves to Identify Children’s Mental Health Problems by One or Two Questions. Sci World J.

[21] Saari A, Sankilampi U, Hannila M-L, Kiviniemi V, Kesseli K, Dunkel L. New Finnish growth references for children and adolescents aged 0 to 20 years: Length/height-for-age, weight-for-length/height, and body mass index-for-age. Ann Med. 2011;43:235–48. 10.3109/07853890.2010.515603 20854213

[22] Rosen DS, American Academy of Pediatrics Committee on Adolescence (2010). Identification and management of eating disorders in children and adolescents. Pediatrics.

[23] Mindell JA, Owens JA, Carskadon MA (1999). Developmental Features of Sleep. Child Adolesc Psychiatr Clin N Am.

[24] Beitchman JH, Young AR (1997). Learning Disorders With a Special Emphasis on Reading Disorders: A Review of the Past 10 Years. J Am Acad Child Adolesc Psychiatry.

[25] Stempel H, Cox-Martin M, Bronsert M, Dickinson LM, Allison MA (2017). Chronic School Absenteeism and the Role of Adverse Childhood Experiences. Acad Pediatr.

[26] Beardselee WR, Versage EM, Giadstone TRG (1998). Children of Affectively Ill Parents: A Review of the Past 10 Years. J Am Acad Child Adolesc Psychiatry.

[27] Häkkänen P, But A, Ketola E, Laatikainen T (2019). Distinct age-related patterns of overweight development to guide school health care interventions. Acta Paediatr.

[28] Baltag V, Levi M. Organizational models of school health services in the WHO European Region. J Health Organ Manag. 2013;27:733–46. 10.1108/JHOM-08-2011-0084. 10.1108/JHOM-08-2011-0084 24422256

[29] Hutson JM, Vikraman J, Li R, Thorup J (2017). Undescended testis: What paediatricians need to know. J Paediatr Child Health.

[30] Newburger JW, Rosenthal A, Williams RG, Fellows K, Miettinen OS (1983). Noninvasive Tests in the Initial Evaluation of Heart Murmurs in Children. N Engl J Med Boston.

[31] Kostopoulou* E, Dimitriou G, Karatza A. Cardiac Murmurs in Children: A Challenge For The Primary Care Physician. Current Pediatric Reviews. 2019;15:131–8. 10.2174/1573396315666190321105536 30907325

[32] Fleming TR (1996). Surrogate End Points in Clinical Trials: Are We Being Misled?. Ann Intern Med.

[33] Krogsbøll LT, Jørgensen KJ, Gøtzsche PC. General health checks in adults for reducing morbidity and mortality from disease. Cochrane Database Syst Rev. 2019. 10.1002/14651858.CD009009.pub3.

[34] Lloyd J, Creanor S, Logan S, Green C, Dean SG, Hillsdon M (2018). Effectiveness of the Healthy Lifestyles Programme (HeLP) to prevent obesity in UK primary-school children: a cluster randomised controlled trial. Lancet Child Adolesc Health.

[35] Häkkänen P, Ketola E, Laatikainen T (2016). Development of overweight and obesity among primary school children—a longitudinal cohort study. Fam Pract.

[36] Council on School Health (2013). Role of the School Physician. Pediatrics.

[37] Council on School Health (2016). Role of the School Nurse in Providing School Health Services. Pediatrics.

[38] Coker TR, Thomas T, Chung PJ (2013). Does Well-Child Care Have a Future in Pediatrics?. Pediatrics.

[39] McPherson M, Arango P, Fox H, Lauver C, McManus M, Newacheck PW (1998). A New Definition of Children With Special Health Care Needs. Pediatrics.

