# Supplementary material for: Associations between study questionnaire-assessed need and school doctor-evaluated benefit of routine health checks: an observational study
Source: BMC Pediatr. 2021 Aug 16;21:346. doi: 10.1186/s12887-021-02810-0 (PMC8365945; doi:10.1186/s12887-021-02810-0)
Supplement: Supplementary file 1 — Additional file 1: Eligible study questionnaires of parents, nurses, and teachers, doctors' electronic reports and parents' and children's PREMS [file 12887_2021_2810_MOESM1_ESM.pdf]

Additional file 1 Eligible study questionnaires of parents, nurses, and teachers, doctors' electronic reports and parents' and children's PREMS

	**Grade 1** n=506, n (%)	**Grade 5** n=507, n (%)	**Total** n=1013, n (%)
**PQ, received**	504 (99.6)	505 (99.6)	1009 (99.6)
Missing date	7 (1.4)	4 (0.8)	11 (1.1)
Late questionnaire	7 (1.4)	5 (1.0)	12 (1.2)
Eligible	497 (98.6)	500 (99.0)	997 (98.8)
**NQ, received**	488 (96.4)	495 (97.6)	983 (97.0)
Missing date	0 (0.0)	3 (0.6)	3 (0.3)
Late questionnaire	9 (1.8)	5 (1.0)	14 (1.4)
Eligible	479 (98.2)	490 (99.0)	969 (98.6)
**TQ, received**	396 (78.3)	419 (82.6)	815 (80.5)
Missing date	14 (3.5)	3 (0.7)	17 (2.1)
Late questionnaire	159 (40.2)	125 (29.8)	284 (34.8)
Eligible	396 (100.0)	419 (100.0)	815 (100.0)
**Doctor's electronic report, received**	506 (100.0)	507 (100.0)	1013 (100.0)
Missing date	1 (0.2)	1 (0.2)	2 (0.2)
Missing evaluation of benefit or harm	2 (0.4)	1 (0.2)	3 (0.3)
Eligible	504 (99.6)	506 (99.8)	1010 (99.7)
**Parent's PREM, received**	495 (97.8)	479 (94.5)	974 (96.2)
Eligible	495 (100.0)	479 (100.0)	974 (100.0)
**Child's PREM, received**	494 (97.6)	497 (98.0)	991 (97.8)
Eligible	494 (100.0)	497 (100.0)	991 (100.0)

Percentages were calculated as follows: Questionnaires received as a percentage of the total number of participants; Missing date, late questionnaires, and eligible questionnaires as a percentage of the questionnaires received. Parents’ and nurses’ study questionnaires were rejected if they were completed too late (after the health check by a doctor). All teachers’ study questionnaires were accepted, because teachers seldom receive information about the child’s health check that would interfere with the study method of collecting the teachers’ concerns before the health check. PQ = parent’s questionnaire, NQ = nurse’s questionnaire, TQ = teacher’s questionnaire, PREM = patient-reported experience measure.
