# Supplementary material for: Associations between study questionnaire-assessed need and school doctor-evaluated benefit of routine health checks: an observational study
Source: BMC Pediatr. 2021 Aug 16;21:346. doi: 10.1186/s12887-021-02810-0 (PMC8365945; doi:10.1186/s12887-021-02810-0)
Supplement: Supplementary file 2 — Additional file 2: Evaluation of the benefit or harm of a doctor’s health check by the doctors, parents, and children [file 12887_2021_2810_MOESM2_ESM.pdf]

Additional file 2 Evaluation of the benefit or harm of a doctor's health check by the doctors, parents, and children

Respondents		**Grade 1**, n (%)	**Grade 5**, n (%)	**Total**, n (%)
**Doctors**	A great deal of benefit	42 (8.3)	46 (9.1)	88 (8.7)
	Quite a lot of benefit	149 (29.6)	173 (34.2)	322 (31.9)
	Only a little benefit	169 (33.5)	167 (33.0)	336 (33.3)
	No benefit or harm	141 (28.0)	117 (23.1)	258 (25.5)
	Only a little harm	3 (0.6)	3 (0.6)	6 (0.6)
	Quite a lot of harm			
**Parents**	A great deal of benefit	203 (41.0)	175 (36.5)	378 (38.8)
	Quite a lot of benefit	217 (43.8)	217 (45.3)	434 (44.6)
	Only a little benefit	60 (12.1)	59 (12.3)	119 (12.2)
	No benefit or harm	15 (3.0)	21 (4.4)	36 (3.7)
	Quite a lot of harm		3 (0.6)	3 (0.3)
	I don't know		4 (0.8)	4 (0.4)
**Children**	Quite a lot of benefit	321 (65.0)	277 (55.7)	598 (60.3)
	Only a little benefit	118 (23.9)	165 (33.2)	283 (28.6)
	No benefit or harm	44 (8.9)	54 (10.9)	98 (9.9)
	Only a little harm	1 (0.2)	1 (0.2)	2 (0.2)
	Quite a lot of harm	10 (2.0)		10 (1.0)
