# Supplementary material for: Associations between study questionnaire-assessed need and school doctor-evaluated benefit of routine health checks: an observational study
Source: BMC Pediatr. 2021 Aug 16;21:346. doi: 10.1186/s12887-021-02810-0 (PMC8365945; doi:10.1186/s12887-021-02810-0)
Supplement: Supplementary file 3 — Additional file 3: Need for a doctor’s health check according to the wish question of the study questionnaires [file 12887_2021_2810_MOESM3_ESM.pdf]

Additional file 3 Need for a school doctor's health check according to the wish question of the study questionnaires

Respondents	**Grade 1**, n (%)	**Grade 5**, n (%)	**Total**, n (%)
**Parents**			
Wish-	194 (42.1)	236 (50.0)	430 (46.1)
Wish+	267 (57.9)	236 (50.0)	503 (53.9)
**Nurses**			
Wish-	183 (42.3)	216 (46.0)	399 (44.2)
Wish+	250 (57.7)	254 (54.0)	504 (55.8)
**Teachers**			
Wish-	289 (79.0)	306 (74.3)	595 (76.5)
Wish+	77 (21.0)	106 (25.7)	183 (23.5)
**Parents and nurses**[a]			
Wish-	129 (25.9)	146 (28.8)	275 (27.3)
Wish+	370 (74.1)	361 (71.2)	731 (72.7)
**Parents, nurses, and teachers**[a]			
Wish-	128 (25.4)	129 (25.4)	257 (25.4)
Wish+	375 (74.6)	378 (74.6)	753 (74.6)

Wish- = "No need for a doctor's health check". Wish+ = "Needs a doctor's health check" and "Consultation with a nurse/doctor may be sufficient" combined. [a]Wish- indicates that none of the respondents had Wish+. Wish+ indicates that at least one of the respondents had Wish+. Please note: Wish-question requires the use of full study questionnaires, because this question refers to all questionnaire responses. For parents, the question read: "Do you wish to speak with the school doctor about these concerns or some other concern related to the child's well-being?" For nurses and teachers, the question read: "Do you wish the school doctor to address these concerns or some other concern related to the well-being of the pupil?".
