# Supplementary material for: Associations between study questionnaire-assessed need and school doctor-evaluated benefit of routine health checks: an observational study
Source: BMC Pediatr. 2021 Aug 16;21:346. doi: 10.1186/s12887-021-02810-0 (PMC8365945; doi:10.1186/s12887-021-02810-0)
Supplement: Supplementary file 4 — Additional file 4: Association of the response to the wish question and benefit of the doctor’s health check; multilevel logistic regression [file 12887_2021_2810_MOESM4_ESM.pdf]

Additional file 4 Association of the response to the wish question and benefit of the doctor's health check; multilevel logistic regression

	**Grade 1**				**Grade 5**				**Total**			
Wish	N	Benefit+ n (%)	OR (95% CI)	P-value	N	Benefit+ n (%)	OR (95% CI)	P-value	N	Benefit+ n (%)	OR (95% CI)	P-value
**Parents**												
Wish-	193	46 (23.8)	1		236	81 (34.3)	1		429	127 (29.6)	1	
Wish+	266	128 (48.1)	3.02 (1.97-4.63)	<0.0001	235	122 (51.9)	2.22 (1.51-3.29)	<0.0001	501	250 (49.9)	2.56 (1.92-3.41)	<0.0001
**Nurses**												
Wish-	183	55 (30.1)	1		216	67 (31.0)	1		399	122 (30.6)	1	
Wish+	249	112 (45.0)	2.04 (1.33-3.13)	0.0011	253	139 (54.9)	2.64 (1.77-3.93)	<0.0001	502	251 (50.0)	2.27 (1.69-3.05)	<0.0001
**Teachers**												
Wish-	289	104 (36.0)	1		306	119 (38.9)	1		595	223 (37.5)	1	
Wish+	77	41 (53.3)	2.42 (1.39-4.22)	0.0020	105	58 (55.2)	2.00 (1.24-3.21)	0.0045	182	99 (54.4)	2.11 (1.47-3.04)	<0.0001
**Parents and nurses**[a]												
Wish-	128	26 (20.3)	1		146	35 (24.0)	1		274	61 (22.3)	1	
Wish+	369	163 (44.2)	3.39 (2.05-5.61)	<0.0001	360	184 (51.1)	3.40 (2.17-5.33)	<0.0001	729	347 (47.6)	3.29 (2.35-4.59)	<0.0001
**Parents, nurses, and teachers**[a]												
Wish-	127	22 (17.3)	1		129	31 (24.0)	1		256	53 (20.7)	1	
Wish+	374	168 (44.9)	4.30 (2.53-7.33)	<0.0001	377	188 (49.9)	3.36 (2.10-5.37)	<0.0001	751	356 (47.4)	3.60 (2.53-5.11)	<0.0001

Wish- = No need for health check by doctor. Wish+ = "Needs health check by doctor" and "Consultation of nurse/doctor may be sufficient" combined. Benefit+ = "Quite a lot or more benefit". Benefit- = "Only a little benefit", "No benefit or harm", "Only a little harm", "Quite a lot of harm" and "I don't know" combined. OR=Odds ratio, CI=Confidence interval. [a]Wish- indicates that none of the respondents had Wish+. Wish+ indicates that at least one of the respondents had Wish+.
